# Urinary Concentration of Monocyte Chemoattractant Protein-1 in Idiopathic Glomerulonephritis: A Long-Term Follow-Up Study

**DOI:** 10.1371/journal.pone.0087857

**Published:** 2014-01-29

**Authors:** Rafid Tofik, Sophie Ohlsson, Omran Bakoush

**Affiliations:** 1 Department of Nephrology, Lund University, Lund, Sweden; 2 Department of Internal Medicine, UAE University, Al-Ain, United Arab Emirates; National Institute for Infectious Diseases (L. Spallanzani), Italy

## Abstract

**Background:**

Monocyte chemoattractant protein-1 (MCP-1), which is up regulated in kidney diseases, is considered a marker of kidney inflammation. We examined the value of urine MCP-1 in predicting the outcome in idiopathic glomerulonephritis.

**Methods:**

Between 1993 and 2004, 165 patients (68 females) diagnosed with idiopathic proteinuric glomerulopathy and with serum creatinine <150 µmol/L at diagnosis were selected for the study. Urine concentrations of MCP-1 were analyzed by ELISA in early morning spot urine samples collected on the day of the diagnostic kidney biopsy. The patients were followed until 2009. The progression rate to end-stage kidney disease was calculated using Kaplan–Meier survival analysis. End-stage kidney disease (ESKD) was defined as the start of kidney replacement therapy during the study follow-up time.

**Results:**

Patients with proliferative glomerulonephritis had significantly higher urinary MCP-1 excretion levels than those with non-proliferative glomerulonephritis (p<0.001). The percentage of patients whose kidney function deteriorated significantly was 39.0% in the high MCP-1 excretion group and 29.9% in the low MCP-1 excretion group. However, after adjustment for confounding variables such as glomerular filtration rate (GFR) and proteinuria, there was no significant association between urine MCP-1 concentration and progression to ESKD, (HR = 1.75, 95% CI = 0.64–4.75, p = 0.27).

**Conclusion:**

Our findings indicate that progression to end-stage kidney disease in patients with idiopathic glomerulopathies is not associated with urine MCP-1 concentrations at the time of diagnosis.

## Introduction

Glomerular diseases, including idiopathic glomerulonephritis, are major causes of end-stage kidney disease (ESKD). Clinically, they are manifested with haematuria, proteinuria and declining kidney function. Histopathologically, they are associated with inflammation and proliferation of the glomerular tissue and enhanced glomerular and interstitial production of inflammatory mediators, such as monocyte chemoattractant protein-1 (MCP-1) [1], [2].

Inflammatory cytokines, including MCP-1, play an important role in glomerular inflammation. MCP-1 is produced by macrophages, vascular endothelial cells, monocytes and fibroblasts. It triggers migration and retention of monocytes and transformation of fibroblasts in the glomeruli [3]–[5].

The urinary concentration of MCP-1 increases with increased activity of glomerular diseases (GN) [6]. It is associated with flares of systemic lupus erythematosus (SLE) and small vessel vasculitis [7], [8]. In diabetic nephropathy, increased urinary MCP-1 is associated with faster disease progression to kidney failure [9]. However, the prognostic utility of urinary MCP-1 in primary proteinuric glomerulonephritis has not received much attention. Therefore, we evaluated the utility of urine MCP-1 as a potential predictor of disease outcome in a longitudinal cohort of patients with idiopathic chronic glomerulonephritis.

## Methods

### Patients

The patients were enrolled in the large glomerular disease investigation program that was conducted at the Nephrology Department, Lund University Hospital, Sweden. The cohort and the controls (healthy blood donors) have been described in detail [10], [11].

Of the patients investigated between August 1993 and February 2004, 189 patients (76 females) had initial serum creatinine <150 µmol/L at the time of kidney biopsy. Of these, urine samples were available from 165 patients (68 females) for MCP-1 analysis. The study was approved by the regional ethical committee of Lund (LU 47-02), and all patients gave informed written consent.

Examination of the biopsies showed or confirmed the following diagnoses: mesangial proliferative glomerulonephritis (n = 64), IgA nephropathy (*n* = 28), membranous glomerulonephritis (*n* = 15), minimal change nephropathy (MCN, *n* = 30), focal segmental glomerulosclerosis (FSGS, *n* = 5), and nephrosclerosis (*n* = 23). We excluded patients with severe kindey failure on admission (serum creatinine >150 µmol/L), diabetic nephropathy, crescentic glomerulonephritis, and other secondary causes such as SLE, and small vessel vasculitis associated with antineutrophil cytoplasmic antibodies (ANCA).

Blood pressure was measured using a mercury sphygmomanometer with the patients in a supine position. The diastolic blood pressure was measured at Korotkoff phase V. Mean arterial blood pressure (MAP) was calculated by adding one third of the pulse pressure to the diastolic blood pressure. The kidney biopsies were evaluated for the percentage of global glomerulosclerosis (GGS) and the extent of tubulo-interstitial damage. Interstitial fibrosis was scored semi quantitatively as 0 (absent), 1 (focal) or 2 (diffuse). All patients were followed regularly at the nephrology outpatient clinic of Lund University Hospital and were on a normal protein diet. Clinical data, including measures of serum creatinine, were collected during the scheduled clinic visits. The patients were followed until the last planned follow-up visit in 2009. The primary end point was end-stage kidney disease (ESKD) defined as the start of kidney replacement therapy.

### Laboratory analysis

Blood samples and the first voided urine specimens were obtained in the morning of the day of the kidney biopsy. The patients had no signs of urinary tract infection in urinalysis examination performed at admission. The urinary albumin-to-creatinine ratio (ACR), measured in a spot morning urine sample, was used as a reliable estimate of the degree of proteinuria [12]. ACR (mg/mmol) is the ratio of urine albumin (mg/L) to urine creatinine (mmole/L), IgG-uria (mg/mmol) is defined as the ratio of urine IgG (mg/L) to urine creatinine (mmole/L), and HC-uria (mg/mmol) is defined as the ratio of urine protein HC (alpha-1 microglobulin) (mg/L) to urine creatinine (mmol/L).

Serum and urine creatinine were determined enzymatically using a Kodak Ektachem 700 XR-C system. Serum and urine albumin, IgG, and urine protein HC (alpha-1 microglobulin) were determined by immunoturbidimetry using a Cobas Mira S system (Roche Inc.) and monospecific rabbit antisera obtained from Dako (Copenhagen, Denmark) [13]–[15]. Urine MCP-1 was measured by an ELISA described in detail earlier [11]. Glomerular filtration rate (GFR) was estimated using the Lund-Malmö formula [16], [17]: eGFR for patients with plasma creatinine (pCr) <150 µmol/L  = e^4.62−0.0112*pCr−0.0124*age+0.339*ln(age)−0.2226 (if female)^.

### Statistical methods

The data are expressed as medians and interquartile ranges. Baseline characteristics of the patients' subgroups were compared with non-parametric Mann-Whitney U Test. Correlation was tested using Spearman's correlation coefficient. Multivariate Cox proportional hazards regression analysis was performed to examine the association of the urine-MCP-1 and IgG-uria with the progression to ESKD. Survival analysis was performed using the Kaplan–Meier method. Log rank test was used to assess differences in survival. All statistics were performed using SPSS software, version 17.0 (Chicago, IL, USA). *P*<0.05 was selected as level of significance. The P-values were adjusted for multiple comparisons using Bonferroni-Holm adjustment method. Patients were divided into low and high MCP-1 groups according to the median urine concentration of MCP-1 at 0.05 mg/mmol creatinine (Tables 1 and 2).

**Table 1:** Baseline characteristics of 165 (68 female) patients with idiopathic chronic glomerulonephritis divided according to the urine MCP-1 concentrations (low <0.05 mg/mmol and high >0.05 mg/mmol).

Variables	Low MCP-1	High MCP-1	P-value
Gender (M/F)	79 (55/24)	86 (42/44)	
Age (years)	48.0 (33.0–55.0)	50.0 (31.8–66.0)	0.26
Follow-up (years)	9.4 (5.9–10.5)	6.4 (3.9–8.9)	0.001*
MAP (mm Hg)	100.0 (93.3–110.0)	106.7 (94.2–116.5)	0.06
S. creatinine (µmol/L)	85.5 (72.0–112.3)	93.0 (73.5–115.0)	0.24
GFR (ml/min/1.73)	72.4 (54.7–92.6)	63.0 (46.4–83.8)	0.02*
S. albumin (g/L)	34.0 (28.0–40.0)	29.0 (19.0–34.0)	<0.001*
IgG-uria (mg/mmol)	3.8 (1.1–7.3)	7.8 (2.7–24.2)	<0.001*
HC-uria (mg/mmol)	0.82 (0.5–1.6)	1.9 (0.7–4.3)	0.001*
ACR (mg/mmol)	53.7 (7.2–150.9)	161.9 (58.6–475.7)	<0.001*

Data are presented as median and inter-quartile range (in parentheses).

MAP =  mean arterial blood pressure; GFR =  glomerular filtration rate; ACR =  urine albumin/creatinine ratio; HC-uria  =  urine alpha-1 microglobulin/creatinine ratio.

* The difference between the groups is statistically significant.

**Table 2:** The follow-up data of 165 (68 female) patients with idiopathic chronic glomerulonephritis divided according to the urine MCP-1 concentrations (low <0.05 mg/mmol and high >0.05 mg/mmol).

Variables	Low MCP-1	High MCP-1	P-value
	*(n = 79)*	*(n = 86)*	
Follow-up years	9.4 (5.9–10.5)	6.4 (3.9–8.9)	0.001*
**Treatment:**			
ACE inhibitors	65 (83.3%)	61 (73.5%)	0.18, ns
Immunosuppressives	18 (23%)	33 (38%)	0.023*
Corticosteroids	18	33	
Calcineurin inhibitors	4	6	
Cyclophosphamide	6	11	
EndGFR ml/min/1.73	63.2 (40.5–86.0)	56.3 (24.0–81.2)	0.25, ns
CV Death	6 (7.8%)	8 (9.6%)	0.14, ns
ESKD	8 (10.4%)	16 (19%)	0.18, ns

Data are presented as number and percentage (in parentheses) and median with inter-quartile range (in parentheses) when appropriate.

ACE  =  angiotensin converting enzyme; End GFR  =  glomerular filtration rate at the study end; CV  =  cardiovascular; ESKD  =  End-stage kidney disease

* The difference between the groups is statistically significant.

## Results

**Figure 1:**
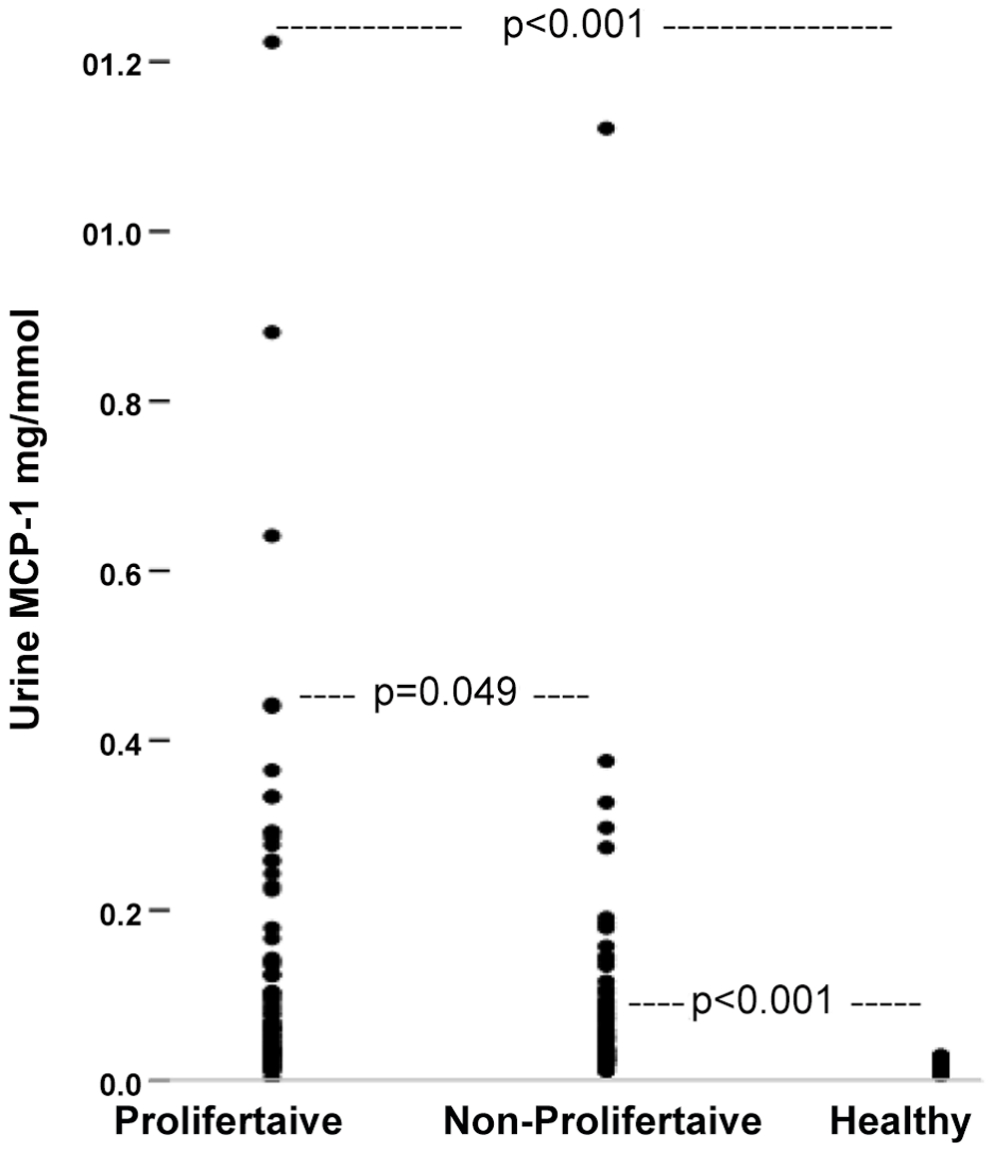
The urine concentrations of MCP-1 in proliferative and non-proliferative forms of glomerulonephritis compared to the urine concentrations of MCP-1 in healthy individuals, *p*<0.001 with Bonferroni-Holm corrections.

Urinary MCP-1 levels were significantly higher in patients with proliferative glomerulonephritis (IgA and mesangioproliferative) than in those with non-proliferative forms of glomerulonephritis (membranous, FSGS, and MCN), (0.061 (IQ 0.032–0.133) mg/mmol creatinine *vs*. 0.039 (IQ 0.027–0.085) mg/mmol creatinine, *p* = 0.049 with Bonferroni-Holm correction; (Figure 1), and both had significantly higher urine MCP-1 concentrations than healthy controls: 0.01 mg/mmol creatinine (IQ 0.008–0.023, *p*<0.001 with Bonferroni-Holm correction (Figure 1). The albuminuria was significantly higher in non-proliferative glomerulonephritis than in proliferative glomerulonephritis (*p* = 0.002). However, there was no statistical difference in the kidney function (*p* = 0.58) or IgG-uria *(p* = 0.33) between proliferative and non-proliferative glomerulonephritis.

During the study, 126 patients were treated with angiotensin converting enzyme inhibitors or angiotensin II receptor antagonist; 51 patients were treated with immunosuppressive drugs (Table 3). There is no difference in the number of patients treated with angiotensin converting enzyme inhibitors in the high and low MCP-1 groups (73.5% *vs* 83.3%, *p* = 0.18; Table 2). However, more patients in the high MCP-1 group were treated with immunosuppressive drugs than those in the low MCP-1 group (46.3% and 25.0%, respectively, *p = 0.008*; Table 2).

**Table 3:** The histological diagnosis and the frequency of treatment with angiotensin converting enzyme inhibitors and immunosuppressive drugs given to 165 (68 female) patients with idiopathic chronic glomerulonephritis.

Condition	N	ACEi	Steroids	Steroids+CPh	Steroids+CI	Total IS
**Mesangio-proliferative GN**	64	46	5	8	4	**17**
**IgA-nephropathy**	28	22	1	2	-	**3**
**MCN**	30	17	9	3	5	**17**
**FSGS**	5	5	1	1	-	**2**
**Membranous nephropathy**	15	15	8	3	1	**12**
**Nephrosclerosis**	23	21	-	-	-	-
**Total**	**165**	**126**	**24**	**17**	**10**	**51**

N  =  number; ACEI  =  angiotensin-converting enzyme inhibitor; CPh  =  cyclophosphamide; CI; calcineurin inhibitors; IS =  immunosuppressive drugs; MCN =  minimal change nephropathy; FSGS =  focal segmental glomerulosclerosis.

Urine MCP-1 was correlated to the degree of IgG-uria, HC-uria and ACR (r = 0.45, 0.38 and 0.41, respectively, *p* = 0.01), but not to the degree of interstitial fibrosis (r = 0.06, p = 0.5). Patients with severe glomerulosclerosis (global sclerosis >20% of glomeruli) had lower urine MCP-1 concentrations than those with a lesser degree of glomerulosclerosis (0.037: IQ 0.026–0.069) *vs*. 0.06: IQ 0.033–0.142, *p* = 0.003).

### Kidney Survival

The kidney function of 32.8% of the patients deteriorated significantly (>3 ml/min/year) during the follow-up time. However, the percentages in the high and the low MCP-1 groups were not significantly different: they were 39% in the high MCP-1 group and 29.9% in the low MCP-1 group (*p* = 0.25, data not shown). Likewise, there was no difference in percentage of patients with deteriorated in kidney function between patients with low or high HC-uria (42.5% *vs*. 27.5%, *p* = 0.1, data not shown), or between patients with low or high ACR (39.5% *vs*. 27.8%, *p* = 0.1, data not shown). On the other hand, during the study follow-up time kidney function deteriorated significantly more frequently (*p* = 0.001) in patients with high IgG-uria (45.5%) than in patients with low IgG-uria (19.4%), data not shown.

**Figure 2:**
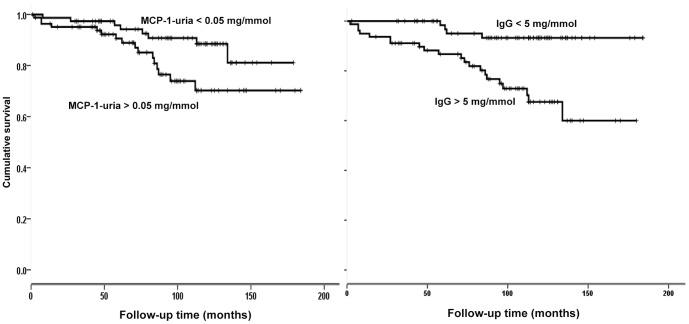
The cumulative risk for end-stage kidney disease according to the IgG-uria and U-MCP-1 in 165 (68 female) patients with idiopathic chronic glomerulonephritis and serum creatinine <150 µmol/L.

During the study follow-up time, 24 patients (9 females) reached ESKD, 14 (4 female) died of cardiovascular events, and 4 (3 females) were lost from follow up (Table 2). Univariate regression analysis revealed that eGFR, urine MCP-1, HC-uria, albuminuria, and IgG-uria are predictors of the outcome of ESKD (Table 4, Figure 2). However, after adjustment for the above-mentioned key confounding variables in a forward conditional stepwise multivariate Cox regression analysis, urine MCP-1 was not significantly associated with the outcome of ESKD (HR = 1.75, 95% CI = 0.64–4.75, *p* = 0.27). Only eGFR and IgG-uria remained as significant predictors for the outcome of ESKD (Table 4, Figure 2).

**Table 4:** Univariate Cox regression analysis for the outcome of end-stage kidney disease in 165 (68 female) patients with idiopathic chronic glomerulnephritis.

Variables	Beta	SE	P-value	HR	95% CI
Age (2gp)	0.33	0.39	0.40	1.39	0.64–3.01
GFR (2gp)	2.59	0.74	<0.001	13.40	3.13–57.44
Albumin-uria (2gp)	0.73	0.41	0.075	2.08	0.93–4.67
HC-uria (2gp)	1.06	0.40	0.008	2.89	1.32–6.35
IgG-uria (2gp)	1.75	0.55	<0.001	5.73	1.96–16.72
MCP-uria (2gp)	0.88	0.44	0.042	2.42	1.03–5.68

2gp =  the cohort was divided into two groups based on the median value of the variable; Beta =  regression coefficient; SE =  standard error; HR =  hazard ratio; CI =  confidence interval.

## Discussion

Mounting evidence shows that urine MCP-1 is a potential diagnostic and prognostic marker in a variety of kidney diseases. Our results confirmed the previous reports of increased urine MCP-1 excretion in patients with glomerulonephritis and its correlation with on-going glomerular inflammation [18]–[20].

Contrary to our expectation, urine MCP-1 was not an independent predictor of long-term outcome in this cohort of patients with idiopathic chronic glomerulonephritis, while IgG-uria was [10]. This seems to contradict previous reports on the prognostic value of urine MCP-1 in patients with systemic diseases [11], [21]–[24]. However, it is more likely that the levels of urine MCP-1 reflect ongoing acute glomerular and tubular inflammation, while IgG-uria reflects the severity of the glomerular damage in both the acute and chronic stages [6], [25].

Proteinuria, including the IgG-uria, is associated with induction of chemokines, accumulation of fibroblasts, and progressive glomerular and interstitial injuries, which ultimately lead to ESKD [26]. Whether or not the glomerular damage is acute or chronic, the glomerular capillary wall loses its size selectivity, allowing leakage of large proteins such as IgG into urine [27]. Many studies have shown that IgG-uria is a powerful predictor of disease outcome in glomerulonephritis [25], [28].

Furthermore, patients with active glomerulonephritis are more likely to receive immune-suppressive therapy. These drugs, as well as the antiproteinuric medications, such as angiotensin converting enzyme inhibitors, block the effect of the inflammatory chemokines and thereby alter the course of glomerulonephritis, diminishing the association between urine MCP-1 level and progression of kidney disease [26], [29], [30].

In our study we measured urine MCP-1 only once, which might have caused misclassification of patients who remitted rapidly and those with repeated flare-ups of glomerulonephritis. However, the cohort was large enough to allow the study of the predictive value of urine biomarkers at the time of diagnosis. Further studies should examine the value of repeated measurement of MCP-1 in idiopathic chronic glomerulonephritis patients, especially for monitoring the treatment response.

In summary, although urine MCP-1 concentration is increased, we found that urine MCP-1 at the time of diagnosis is not a powerful predictor of long-term kidney disease outcome in patients with idiopathic glomerulonephritis. Future studies are needed to investigate the utility of repeated measurement of urine MCP-1 in patients with idiopathic chronic glomerulonephritis.
